# Epidemic Analysis of Wireless Rechargeable Sensor Networks Based on an Attack–Defense Game Model

**DOI:** 10.3390/s21020594

**Published:** 2021-01-15

**Authors:** Guiyun Liu, Baihao Peng, Xiaojing Zhong

**Affiliations:** 1 School of Mechanical and Electric Engineering, Guangzhou University, Guangzhou 510006, China; liugy@gzhu.edu.cn (G.L.); zhongxj@gzhu.edu.cn (X.Z.); 2 School of Electronics and Communication Engineering, Guangzhou University, Guangzhou 510006, China

**Keywords:** wireless rechargeable sensor network, cyber security, stability analysis, optimal control

## Abstract

Energy constraint hinders the popularization and development of wireless sensor networks (WSNs). As an emerging technology equipped with rechargeable batteries, wireless rechargeable sensor networks (WRSNs) are being widely accepted and recognized. In this paper, we research the security issues in WRSNs which need to be addressed urgently. After considering the charging process, the activating anti-malware program process, and the launching malicious attack process in the modeling, the susceptible–infected–anti-malware–low-energy–susceptible (SIALS) model is proposed. Through the method of epidemic dynamics, this paper analyzes the local and global stabilities of the SIALS model. Besides, this paper introduces a five-tuple attack–defense game model to further study the dynamic relationship between malware and WRSNs. By introducing a cost function and constructing a Hamiltonian function, the optimal strategies for malware and WRSNs are obtained based on the Pontryagin Maximum Principle. Furthermore, the simulation results show the validation of the proposed theories and reveal the influence of parameters on the infection. In detail, the Forward–Backward Sweep method is applied to solve the issues of convergence of co-state variables at terminal moment.

## 1. Introduction

**Figure 1:**
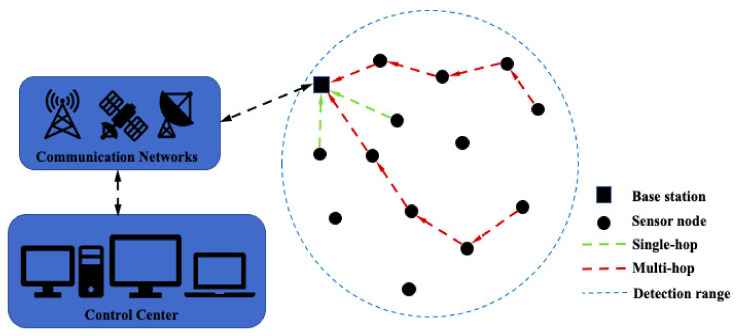
Communication architecture of wireless sensor networks.

Wireless sensor networks (WSNs) are the research hotspot worldwide over the last few years [1,2,3]. Sensor nodes which serve the function of data storing and data transmitting capacities form WSNs in the way of multi-hop or single-hop, as depicted in Figure 1. To monitor the physical parameters, such as temperature, humidity, pressure, etc., sensor nodes are randomly deployed in unattended areas. WSNs have widespread applications which are ranging from everyday life to various manufacturing industries [4]. However, due to the vulnerability of the sensor nodes and battery capacity limitations, the issues of security [5] and short lifespan [6] of WSNs are urgent to be tackled.

Focusing on optimizing energy utilization, scholars have proposed efficient schemes. However, comparing with the optimizing strategies, the operation of deploying rechargeable batteries can figure out the energy problem radically. Networks which are composed of rechargeable sensor nodes are named as wireless rechargeable sensor networks (WRSNs). Research hotspots on WRSNs mainly focus on solving the problems of both charging scheduling and system performance optimizations [7,8,9] in recent years. However, security issues in WRSNs are seldom attracting the attention of scholars. Malware, as a self-replicating malicious code, can lead to network interruption and paralysis once it propagates in the networks. Even worse, rechargeable sensor nodes also suffer from the Denial of Charge (DOC) attacks [10]. Such attacks will cause catastrophic consequence to real-time and pre-warning application fields [11]. Thus, it is urgent to study the security of WRSNs based on the rechargeable characteristics.

For the past few years, some scholars have made contributions to security issues of WRSNs based on the characteristics of information transmission. Recent relevant studies are listed in Table 1.

Due to the high similarity between infection mechanism of diseases in the population and the propagation mechanism of malware in WSNs, epidemic dynamics has also been widely used in the research of WSN security issues. In general, the applications of epidemic dynamics in WSNs mainly focus on the stability analysis of the built model. Recent relevant studies are listed in Table 2.

Although the above models consider the characteristics of WSNs from various aspects, they do not analyze and model the networks based on the energy level. Besides, to our knowledge, the studies combining epidemic dynamics with WRSNs are very few. Therefore, this paper divides sensor nodes in WRSNs according to the residual energy and infection of sensor nodes and introduces the charging process. Differential games are also widely used in WSNs as a method of studying optimal dynamic strategies. Recent relevant studies are listed in Table 3.

Based on the previous works [41] and inspired by [23], this paper proposes an epidemic model that includes the anti-malware (A) state, constructs game between malware and WRSNs, and obtains the optimal control strategies for both parties.

In the research on the security of WRSNs, few scholars analyze the issues by applying the relevant knowledge of epidemic dynamics. By establishing the dynamic differential equations of the propagation of malware in WRSNs, both the propagation mechanism of malware and the defense mechanism of WRSNs can be dynamically understood so as to provide novel thoughts and directions for resisting the invasion of malware.

In this paper, a susceptible infected anti-malware low-energy susceptible (SIALS) model is proposed by considering the charging process and the process of activating of the anti-malware program.The SIALS model can not only reflect the infection in WRSNs but also reveal the trend of the residual energy of the sensor nodes. At the same time, to describe the attack modes of malware, this paper considers the hardware attacks launched by malware and charging process compromised with malware.

Additionally, through the theory of stability analysis, the local and global stabilities of the disease-free equilibrium point and the epidemic equilibrium point of SIALS model are proved. Furthermore, this paper analyzes the game composed of malware and WRSNs by applying the Pontryagin Maximum Principle and obtains the optimal control strategies. Consequently, this work enriches the application of epidemic dynamics and differential games in addressing the security issues on WRSNs.

The rest of the paper is organized as follows. The introduction of the modeling of SIALS is presented in Section 2. Theorems of the local and global stability and the optimal strategies are proved in Section 3. The simulation results are shown in Section 4. The conclusions are drawn in Section 5.

## 2. Modeling

### 2.1. Dynamic Equation

**Figure 2:**
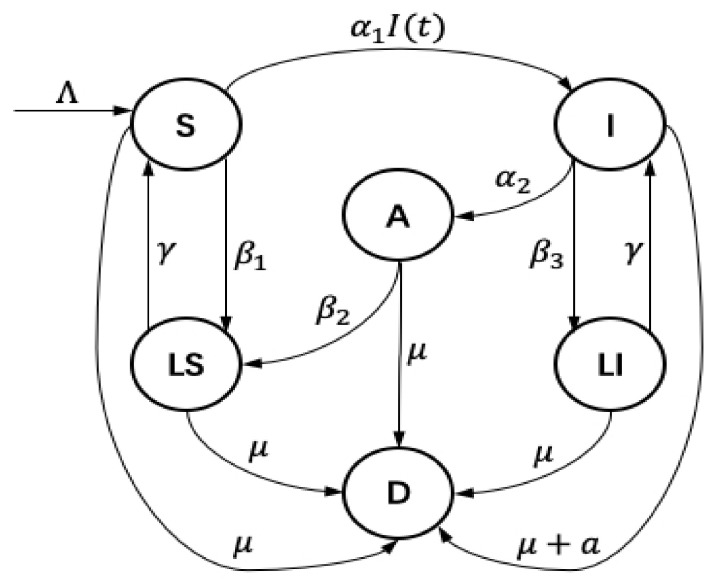
Flow diagram of the improved epidemic model.

In this paper, WRSNs consist of homogeneous rechargeable nodes which are randomly distributed. Meanwhile, the number of nodes increase at rate Λ, where Λ is greater than 0. Suppose that nodes in the networks belong to one of six possible compartment: susceptible (*S*), infected (*I*), anti-malware (*A*), low-energy and susceptible (LS), low-energy and infected (LI), and dysfunction (*D*). The relationship between the six compartments are depicted in Figure 2. *S* nodes are vulnerable to malware; *I* nodes are compromised with attacker; *A* nodes clear malware by activating anti-malware program; LS and LI nodes are both in low-energy level and remain dormant; and *D* nodes are totally out of function. Now, let us impose a set of hypotheses as follows.

(a) Malware propagates by broadcasting. Assuming that the ratio of *I* nodes successfully infecting *S* nodes is $\alpha_{1}S\left(t\right)$, where $\alpha_{1}$ is greater than 0, then the proportion of the new infected in the network is $\alpha_{1}S\left(t\right)I\left(t\right)$.
(b) Considering mobile chargers and rechargeable modules, after the nodes in *A* drop to LS at $\beta_{2}$, anti-malware programs stop running, and the nodes return to *S* at rate γ when they are fully charged. $\beta_{2}$ and γ are all greater than 0.
(c) Nodes in *S*, *I*, and *A* drop to low-energy level at different ratios $\beta_{1}$, $\beta_{3}$, and $\beta_{2}$, where $\beta_{1}<\beta_{2}<\beta_{3}$. Among them, owing to the running of anti-malware program, $\beta_{2}$ is greater than $\beta_{1}$. Due to the software attack launched by malware, $\beta_{3}$ is greater than $\beta_{1}$ and $\beta_{2}$. $\beta_{1}$, $\beta_{2}$, and $\beta_{3}$ are all greater than 0.
(d) Suppose that, except for *I*, the four remaining compartments *S*, *A*, LS, and LI have the same mortality μ. *I* is different in that malware also launches hardware attacks at rate *a* to cause damage. μ and *a* are all greater than 0.
(e) Regardless of other protective measures, this paper only considers activating anti-malware program to achieve the purpose of clearing malware temporarily.

In particular, the parameters are summarized in Table 4.

On the basis of the above hypotheses, a novel dynamical system is obtained in (1)–(6):

(1) $$\dot{S(t)}=\Lambda -\left(\alpha_{1}I\left(t\right)+\beta_{1}+\mu \right)S\left(t\right)+\gamma LS\left(t\right),$$

(2) $$\dot{I(t)}=\alpha_{1}S\left(t\right)I\left(t\right)-\left(\alpha_{2}+\beta_{3}+\mu +a\right)I\left(t\right)+\gamma LI\left(t\right),$$

(3) $$\dot{A(t)}=-\left(\beta_{2}+\mu \right)A\left(t\right)+\alpha_{2}I\left(t\right),$$

(4) $$\dot{LI(t)}=-\left(\gamma +\mu \right)LI\left(t\right)+\beta_{3}I\left(t\right),$$

(5) $$\dot{LS(t)}=-\left(\gamma +\mu \right)LS\left(t\right)+\beta_{1}S\left(t\right)+\beta_{2}A\left(t\right),$$

and

(6) $$\dot{D(t)}=\mu N\left(t\right)+aI\left(t\right),$$

where N(t)=S(t)+I(t)+A(t)+LS(t)+LI(t) and

(7) $$\dot{N(t)}=\Lambda -\mu N\left(t\right)-aI\left(t\right).$$

### 2.2. Computation of the Steady States and the Basic Reproductive Number

Considering LS(t)=N(t)−S(t)−I(t)−A(t)−LI(t), (1) can be rewritten as

(8) $$\dot{S(t)}=\Lambda -\left(\alpha_{1}I\left(t\right)+\beta_{1}+\mu \right)S\left(t\right)+\gamma \left(N-S\left(t\right)-I\left(t\right)-A\left(t\right)-LI\left(t\right)\right),$$

where $N\left(t\right)=N\left(\infty \right)=\frac{\Lambda -aI(t)}{\mu }$.

Then, the solutions of the limit system (8) and (2)–(4) are the steady states of the system (1)–(5).

The first solution is the disease-free steady state: $E_{0}=\left(S_{0},I_{0},A_{0},LI_{0}\right)$, where $I_{0}=0$, $A_{0}=0$, $LI_{0}=0$, and

(9) $$S_{0}=\frac{\Lambda (\mu +\gamma )}{\left(\mu +\gamma \right)\left(\mu +\beta_{1}\right)-\gamma \beta_{1}}.$$

The second solution is the epidemic steady state $E^{*}=\left(S^{*},I^{*},A^{*},LI^{*}\right)$, and

(10) $$S^{*}=\frac{\left(\alpha_{2}+\beta_{3}+\mu +a\right)\left(\gamma +\mu \right)-\gamma \beta_{3}}{\alpha_{1}\left(\gamma +\mu \right)},$$

(11) $$I^{*}=\frac{\Delta_{1}+\gamma \Lambda \left(\beta_{2}+\mu \right)\left(\gamma +\mu \right)}{\Delta_{2}+\Delta_{3}},$$

(12) $$A^{*}=\frac{\alpha_{2}\Delta_{1}+\alpha_{2}\gamma \Lambda \left(\beta_{2}+\mu \right)\left(\gamma +\mu \right)}{\left(\beta_{2}+\mu \right)\left(\Delta_{2}+\Delta_{3}\right)},$$

and

(13) $$LI^{*}=\frac{\beta_{3}\Delta_{1}+\beta_{3}\gamma \Lambda \left(\beta_{2}+\mu \right)\left(\gamma +\mu \right)}{\left(\gamma +\mu \right)\left(\Delta_{2}+\Delta_{3}\right)},$$

where

(14) $$\Delta_{1}=\left[\Lambda -\left(\beta_{1}+\mu +\gamma \right)S^{*}\right]\left[\mu \left(\beta_{2}+\mu \right)\left(\gamma +\mu \right)\right],$$

(15) $$\Delta_{2}=\mu \left(\beta_{2}+\mu \right)\left(\gamma +\mu \right)\left[\gamma +\alpha_{1}S^{*}\right],$$

and

(16) $$\Delta_{3}=\gamma \left[a\left(\beta_{2}+\mu \right)\left(\gamma +\mu \right)+\alpha_{2}\mu \left(\gamma +\mu \right)+\beta_{3}\mu \left(\beta_{2}+\mu \right)\right].$$

Consequently, considering the next generation matrix method, the basic reproductive number $R_{0}$ is its spectral radius.

Set

(17) $$F=\left(\begin{array}{cc} \alpha_{1}S\left(t\right) & 0\\ 0 & 0 \end{array}\right)$$

and

(18) $$V=\left(\begin{array}{cc} \alpha_{2}+\beta_{3}+\mu +a & -\gamma \\ -\beta_{3} & \gamma +\mu \end{array}\right).$$

Thus,

(19) $$\begin{array}{cc} R_{0}=F\cdot V^{-1}= & \frac{\alpha_{1}S_{0}\left(\gamma +\mu \right)}{\left(\alpha_{2}+\beta_{3}+\mu +a\right)\left(\gamma +\mu \right)-\gamma \beta_{3}}\\ = & \frac{\alpha_{1}\Lambda {\left(\gamma +\mu \right)}^{2}}{\left[\left(\beta_{1}+\mu \right)\left(\gamma +\mu \right)-\gamma \beta_{1}\right]\left[\left(\alpha_{2}+\beta_{3}+\mu +a\right)\left(\gamma +\mu \right)-\gamma \beta_{3}\right]}. \end{array}$$

## 3. Dynamic Analysis and Optimal Strategy

In this section, the stability and the optimal strategy in the SIALS model are discussed. In Section 3.1, the local and global stabilities of the disease-free point are proved by using the eigenvalues and the Lyaponov function. In Section 3.2, the local and global stabilities of the epidemic point are proved by using the Routh criterion and Bendixson-Dulac criterion. In Section 3.3, a five-tuple attack–defense game is proposed and the optimal strategies of malware and WRSNs are obtained by applying the Pontryagin Maximum Principle.

### 3.1. Analysis of Disease-Free Equilibrium Point

**Theorem** **1.** *The disease-free equilibrium point, $E_{0}$, is locally asymptotically stable if $R_{0}<1$.*

**Proof.**  Here, we use matrix eigenvalues to verify the validity of the theorem. In general, if the eigenvalues of the system matrix are negative, then the system must be stable.

Consider the follow matrix

(20) $$F-V=\left(\begin{array}{cc} \alpha_{1}S_{0}-\left(\alpha_{2}+\beta_{3}+\mu +a\right) & \gamma \\ \beta_{3} & -\gamma -\mu \end{array}\right)$$

The eigenvalues of (20) are

(21) $$\lambda_{1}=0.5\left(-B_{1}+\sqrt{B_{1}^{2}+4B_{2}}\right)$$

and

(22) $$\lambda_{2}=0.5\left(-B_{1}-\sqrt{B_{1}^{2}+4B_{2}}\right),$$

where $B_{1}=\left(\gamma +\mu \right)-\left[\alpha_{1}S_{0}-\left(\alpha_{2}+\mu +\beta_{3}+a\right)\right]$ and $B_{2}=\left[\left(\mu +\gamma \right)\left(\alpha_{2}+\beta_{3}+\mu +a\right)-\gamma \beta_{3}\right]\left(R_{0}-1\right)$. The real parts of the two eigenvalues are negative if $R_{0}<1$. Besides,

(23) $$\frac{\partial \left(\Lambda -\left(\beta_{1}+\mu \right)S\left(t\right)+\gamma \left(N-S\right)\right)}{\partial S}=-\beta_{1}-\mu -\gamma <0.$$

Thus, $E_{0}$ is locally asymptotically stable [42] when $R_{0}<1$. Conversely, $E_{0}$ is unstable if $R_{0}>1$. □

**Theorem** **2.** *The disease-free equilibrium point, $E_{0}$, is globally asymptotically stable if $R_{0}\leq 1$.*

**Proof.**  Here, Lyapunov stability method is applied. In general, a positive definite Lyaponov function with negative definite first derivative needs to be established to test the stability of the system [43]. Considering a Lyaponov function V(t)=(γ+μ)I(t)+γLI(t)>0, we have:

(24) $$\begin{array}{cc} \dot{V(t)} & =\left(\gamma +\mu \right)\dot{I(t)}+\gamma \dot{LI(t)}\\ & \leq I\left(t\right)[\left(\gamma +\mu \right)\alpha_{1}S_{0}-\left(\gamma +\mu \right)\left(\alpha_{2}+\beta_{3}+\mu +a\right)+\gamma \beta_{3}]\\ & =\left(\gamma +\mu \right)\left[\alpha_{1}S_{0}I\left(t\right)-\left(\alpha_{2}+\beta_{3}+\mu +a\right)I\left(t\right)\right]+\gamma \beta_{3}I\left(t\right)\\ & =I\left(t\right)[\left(\gamma +\mu \right)\alpha_{1}S_{0}-\left(\gamma +\mu \right)\left(\alpha_{2}+\beta_{3}+\mu +a\right)+\gamma \beta_{3}]\\ & \leq I\left(t\right)\left(R_{0}-1\right) \end{array}$$

In addition, $\frac{dV}{dt}=0$ if and only if $R_{0}=1$ and I(t)=0. Moreover, (*S*, *I*, *A*, LI) tends to $E_{0}$ when *t* tends to infinity, and the maximum invariant set in $\left\{\left(S,I,A,LI\right)\in \Omega :\frac{dV}{dt}=0\right\}$ is $E_{0}$. Thus, Theorem 2 is proved, after considering the La-Salle Invariance Principle [44]. □

### 3.2. Analysis of Epidemic Equilibrium Point

**Theorem** **3.** The epidemic equilibrium point, $E^{*}$, is locally asymptotically stable if $R_{0}>1$.

**Proof.**  Here, the Routh criterion is applied to prove the theorem. Firstly, the Jacobian matrix of the limit system is:

(25) $$\left(\begin{array}{cccc} -(\alpha_{1}I\left(t\right)+\beta_{1}+\mu )-\gamma & -\alpha_{1}S\left(t\right)-\gamma -\frac{a\gamma }{\mu } & -\gamma & -\gamma \\ \alpha_{1}I\left(t\right) & \alpha_{1}S\left(t\right)-\left(\alpha_{2}+\beta_{3}+\mu +a\right) & 0 & \gamma \\ 0 & \alpha_{2} & -(\beta_{2}+\mu ) & 0\\ 0 & \beta_{3} & 0 & -(\gamma +\mu ) \end{array}\right)$$

Then, the characteristic polynomial of (25) in $E^{*}$ is

(26) $$P\left(\lambda \right)=P_{1}\lambda^{4}+P_{2}\lambda^{3}+P_{3}\lambda^{2}+P_{4}\lambda^{1}+P_{5},$$

where

(27) $$P_{1}=1>0,$$

(28) $$P_{2}=a+\beta_{1}+\alpha_{2}+\beta_{2}+\beta_{3}+2\gamma +4\mu +\alpha_{1}\theta_{3}\left(R_{0}-1\right)+\frac{\gamma \beta_{3}}{\gamma +\mu }>0,$$

(29) $$P_{3}=\left(\beta_{2}+\mu \right)\frac{\gamma \beta_{3}}{\gamma +\mu }+\alpha_{1}\left(\alpha_{1}S^{*}+\gamma \right)\theta_{3}\left(R_{0}-1\right)+\left(\gamma +\mu \right)\left(\beta_{2}+\mu +\frac{\gamma \beta_{3}}{\gamma +\mu }\right)+\theta_{1}\left(\theta_{2}+\frac{\gamma \beta_{3}}{\gamma +\mu }\right)>0,$$

(30) $$P_{4}=\left(\gamma +\mu \right)\left(\beta_{2}+\mu \right)\frac{\gamma \beta_{2}}{\gamma +\mu }+\alpha_{1}\theta_{3}\left(R_{0}-1\right)\left(\alpha_{1}S^{*}+\gamma \right)\left(\beta_{2}+\mu \right)\theta_{2}+\theta_{1}\left[\left(\gamma +\mu \right)\left(\beta_{2}+\mu \right)+\theta_{2}\frac{\gamma \beta_{3}}{\gamma +\mu }\right]>0,$$

and

(31) $$P_{5}=\alpha_{1}\theta_{3}\left(R_{0}-1\right)\left(\alpha_{1}S^{*}+\gamma \right)\left(\gamma +\mu \right)\left(\beta_{2}+\mu \right)>0,$$

where

(32) $$\theta_{1}=\alpha_{1}I^{*}+\beta_{1}+\mu +\gamma ,$$

(33) $$\theta_{2}=\gamma +2\mu +\beta_{2},$$

and

(34) $$\theta_{3}=\frac{\beta_{2}+\mu }{\alpha_{1}\left(\gamma +\mu \right)\left[\left(\alpha_{2}+\beta_{3}+\mu +a\right)+\gamma \mu \left(\beta_{2}+\mu \right)+a\gamma \left(\beta_{2}+\mu \right)+\alpha_{2}\gamma \mu \right]}$$

Moreover, a simple calculation shows $P_{2}P_{3}-P_{1}P_{4}>0$ and $P_{2}P_{3}P_{4}-P_{1}P_{4}^{2}-P_{2}^{2}P_{5}>0$. Thus, if $R_{0}>1$, applying the Routh criterion [45], the local asymptotically stability of $E^{*}$ is tenable. □

**Theorem** **4.** The epidemic equilibrium point, $E^{*}$, is globally asymptotically stable if $R_{0}\geq 1$.

**Proof.**  Set

(35) $$D\left(I,LI\right)=\frac{1}{ILI},$$

(36) $$P=\alpha_{1}S\left(t\right)I\left(t\right)-\left(\alpha_{2}+\beta_{2}+\mu +a\right)I\left(t\right)+\gamma LI\left(t\right),$$

and

(37) $$Q=-\left(\gamma +\mu \right)LI\left(t\right)+\beta_{3}I\left(t\right).$$

Considering the following formulation:

(38) $$\frac{\partial \left(DP\right)}{\partial I}+\frac{\partial \left(DQ\right)}{\partial LI}=-\gamma I^{-2}-\beta_{3}LI^{-2}<0$$

By applying the Bendixson–Dulac criterion [46], the system admits no periodic orbits in the interior of Ω.

Let (*I*, *LI*) be a smooth point on the boundary of Ω. Along the boundary, there exists two possibilities:

(a) 0≤I<1, LI=0. Then, $\frac{dP(t)}{dt}=\beta_{3}I\left(t\right)\geq 0$. The value 0 occurs if and only if I=0.
(b) 0≤LI<1, I=0. Then, $\frac{dQ(t)}{dt}=\gamma LI\left(t\right)\geq 0$. The value 0 occurs if and only if LI=0.

Thus, there is no periodic solutions that pass through the boundary.

In view of Theorem 3, the claim follows from the generalized Poincare–Bendixson theorem [46]. □

### 3.3. Optimal Strategies

Based on the evolution of node state during the confrontation between malware and WRSNs, an attack–defense game model is constructed as follows.

The attack–defense game based on the SIALS model can be expressed as a five-tuple G={P,ν,μ,X,Λ}, where

- $P=\{P_{A},P_{D}\}$ is the set of plays in the attack–defense game. $P_{A}$ is the attacker and $P_{D}$ is the defender.
- $\nu =\{A_{SI}\left(t\right),A_{LII}\left(t\right),A_{ID}\left(t\right)\}$ is a set of strategies implemented by the malware. $A_{SI}\left(t\right)$ represents the spreading capability of the malware, $A_{LII}\left(t\right)$ represents the strength of the attacks on the charging process, and $A_{ID}\left(t\right)$ represents the strength of the hardware attack. In particular, the three control strategies are all constrained by the upper and lower bounds.
- $\mu =\{D_{IA}\left(t\right),D_{LSS}\left(t\right)\}$ is a set of strategies implemented by the WRSNs. $D_{IA}\left(t\right)$ represents the strength of activation of the anti-malware program and $D_{LSS}\left(t\right)$ represents the control of the charging process by WRSNs. Similarly, the two strategies have upper and lower bounds.
- X={X(t)|S(t),I(t),A(t),LS(t),LI(t),D(t)} is a set of the state variables on the SIALS model. The denotations of the state variables are the same as the statement in Section 2.1.
- $\Lambda =\{\Lambda \left(t\right)|\lambda_{S}\left(t\right),\lambda_{I}\left(t\right),\lambda_{A}\left(t\right),\lambda_{LS}\left(t\right),\lambda_{LI}\left(t\right),\lambda_{D}\left(t\right)\}$ is a set of the adjoint variables of the games

Considering the controlled process stated above, (1)–(6) transform to

(39) $$\dot{S}\left(t\right)=\Lambda -\left(\alpha_{1}A_{SI}\left(t\right)I\left(t\right)+\beta_{1}+\mu \right)S\left(t\right)+\gamma D_{LSS}\left(t\right)LS\left(t\right),$$

(40) $$\dot{I}\left(t\right)=\alpha_{1}A_{SI}\left(t\right)S\left(t\right)I\left(t\right)-\left(\alpha_{2}D_{IA}\left(t\right)+\beta_{3}+\mu +aA_{ID}\left(t\right)\right)I\left(t\right)+\gamma A_{LII}\left(t\right)LI\left(t\right),$$

(41) $$\dot{A(t)}=-\left(\beta_{2}+\mu \right)A\left(t\right)+\alpha_{2}D_{IA}\left(t\right)I\left(t\right),$$

(42) $$\dot{LS(t)}=-\left(\gamma D_{LSS}\left(t\right)+\mu \right)LS\left(t\right)+\beta_{1}S\left(t\right)+\beta_{2}A\left(t\right),$$

(43) $$\dot{LI(t)}=-\left(\gamma A_{LII}\left(t\right)+\mu \right)LI\left(t\right)+\beta_{3}I\left(t\right),$$

and

(44) $$\dot{D(t)}=\mu N\left(t\right)+aA_{ID}\left(t\right)I\left(t\right).$$

In this paper, we mainly focus on how to effectively suppress the growth of malware. Furthermore, in the purpose of maintaining the operation of the networks, the phenomenon of network interruption and paralysis caused by the dysfunctionality of the sensor nodes need to be minimized. Therefore, the number of the infected and dysfunctional sensor nodes is used to measure the overall cost in the attack–defense game. Set J(·) as the overall cost of the game and

(45) $$J\left(X\left(t\right),\mu \left(t\right),\nu \left(t\right)\right)=\int_{t_{0}}^{t_{f}}\left\{C_{I}I\left(t\right)+C_{D}D\left(t\right)\right\}dt.$$

The above description of the cost index is a classic Lagrange problem in differential games. In (6), $t_{0}$ and $t_{f}$, respectively, represent the initial and terminal moment of the game. Specifically, $C_{I}I\left(t\right)$ is the instantaneous cost determined by the damage capability and the number of *I* nodes at time *t*, where $C_{I}>0$. $C_{D}D\left(t\right)$ is the instantaneous cost determined by the impact of network interruption and paralysis at time *t*, where $C_{D}>0$.

In this game, the goal of both parties is to influence changes in the cost J(·) to make it more beneficial to their own development. Malware aims to maximize J(·), while WRSNs aim to minimize J(·). Therefore, malware needs to apply the dynamic strategies in ν(t) to maximize J(·) and WRSNs need to use the dynamic strategies in μ(t) to minimize the J(·). To achieve the purpose of both parties, Theorem 5 is given by applying the Pontryagin Maximum Principle.

**Theorem** **5.** Based on the state functions (39)–(44), there exist an optimal strategy set $\left\{\mu^{*}\left(t\right),\nu^{*}\left(t\right)\right\}=\left\{\left(D_{IA}^{*}\left(t\right),D_{LSS}^{*}\left(t\right)\right),\left(A_{SI}^{*}\left(t\right),A_{ID}^{*}\left(t\right),A_{LII}^{*}\left(t\right)\right)\right\}$ in the attack–defense game such that

(46) $$J\left(X\left(t\right),\mu^{*}\left(t\right),\nu^{*}\left(t\right)\right)=max_{\nu }min_{\mu }J\left(X\left(t\right),\mu \left(t\right),\nu \left(t\right)\right)=min_{\mu }max_{\nu }J\left(X\left(t\right),\mu \left(t\right),\nu \left(t\right)\right).$$

*The expressions of the optimal strategies are*

(47) $$A_{SI}^{*}\left(t\right)=\left\{\begin{array}{ccc} maxA_{SI}, & \; & \left(\lambda_{I}\left(t\right)-\lambda_{S}\left(t\right)\right)\alpha_{1}S\left(t\right)I\left(t\right)>0\\ minA_{SI}, & \; & \left(\lambda_{I}\left(t\right)-\lambda_{S}\left(t\right)\right)\alpha_{1}S\left(t\right)I\left(t\right)<0, \end{array}\right.$$

(48) $$A_{ID}^{*}\left(t\right)=\left\{\begin{array}{ccc} maxA_{ID}, & \; & (\lambda_{D}\left(t\right)-\lambda_{I}\left(t\right))aI\left(t\right)>0\\ minA_{ID}, & \; & (\lambda_{D}\left(t\right)-\lambda_{I}\left(t\right))aI\left(t\right)<0, \end{array}\right.$$

(49) $$A_{LII}^{*}\left(t\right)=\left\{\begin{array}{ccc} maxA_{LII}, & \; & \left(\lambda_{I}\left(t\right)-\lambda_{LI}\left(t\right)\right)\gamma LI\left(t\right)+C_{C}\gamma LI\left(t\right)>0\\ minA_{LII}, & \; & \left(\lambda_{I}\left(t\right)-\lambda_{LI}\left(t\right)\right)\gamma LI\left(t\right)+C_{C}\gamma LI\left(t\right)<0, \end{array}\right.$$

(50) $$D_{IA}^{*}\left(t\right)=\left\{\begin{array}{ccc} minD_{IA}, & \; & \left(\lambda_{A}\left(t\right)-\lambda_{I}\left(t\right)\right)\alpha_{2}I\left(t\right)>0\\ maxD_{IA}, & \; & \left(\lambda_{A}\left(t\right)-\lambda_{I}\left(t\right)\right)\alpha_{2}I\left(t\right)<0, \end{array}\right.$$

*and*

(51) $$D_{LSS}^{*}\left(t\right)=\left\{\begin{array}{ccc} minD_{LSS}, & \; & \left(\lambda_{S}\left(t\right)-\lambda_{LS}\left(t\right)\right)\gamma LS\left(t\right)+C_{C}\gamma LS\left(t\right)>0\\ maxD_{LSS}, & \; & \left(\lambda_{S}\left(t\right)-\lambda_{LS}\left(t\right)\right)\gamma LS\left(t\right)+C_{C}\gamma LS\left(t\right)<0. \end{array}\right.$$

**Proof.**  First, there exists a saddle-point in the game according to [41].

Then, in view of (39)–(44) and (45), the Hamiltonian function constructs as:

(52) $$\begin{array}{cc} H(X(t),\lambda (t),\mu (t),\nu (t),t)= & \lambda_{S}\left(t\right)\dot{S(t)}+\lambda_{I}\left(t\right)\dot{I(t)}+\lambda_{A}\left(t\right)\dot{A(t)}+\lambda_{LS}\left(t\right)\dot{LS(t)}\\ & +\lambda_{LI}\left(t\right)\dot{LI(t)}+\lambda_{D}\left(t\right)\dot{D(t)}+C_{I}I\left(t\right)+C_{D}D\left(t\right) \end{array}$$

Note that the constraints of the adjoint variables are given by the following formulas [44]:

(53) $$\dot{\lambda_{S}\left(t\right)}=\left(\lambda_{S}\left(t\right)-\lambda_{I}\left(t\right)\right)\alpha_{1}A_{SI}\left(t\right)I\left(t\right)+\left(\lambda_{S}\left(t\right)-\lambda_{LS}\left(t\right)\right)\beta_{1}+\left(\lambda_{S}\left(t\right)-\lambda_{D}\left(t\right)\right)\mu ,$$

(54) $$\begin{array}{cc} \dot{\lambda_{I}\left(t\right)}= & \left(\lambda_{S}\left(t\right)-\lambda_{I}\left(t\right)\right)\alpha_{1}A_{SI}\left(t\right)S\left(t\right)+\left(\lambda_{I}\left(t\right)-\lambda_{A}\left(t\right)\right)\alpha_{2}D_{IA}\left(t\right)\\ & +\left(\lambda_{I}\left(t\right)-\lambda_{LI}\left(t\right)\right)\beta_{3}+\left(\lambda_{I}\left(t\right)-\lambda_{D}\left(t\right)\right)\left(\mu +aA_{ID}\left(t\right)\right)-C_{I}, \end{array}$$

(55) $$\dot{\lambda_{A}\left(t\right)}=\left(\lambda_{A}\left(t\right)-\lambda_{LS}\left(t\right)\right)\beta_{2}+\left(\lambda_{A}\left(t\right)-\lambda_{D}\left(t\right)\right)\mu ,$$

(56) $$\dot{\lambda_{LS}\left(t\right)}=\left(\lambda_{LS}\left(t\right)-\lambda_{S}\left(t\right)\right)\gamma D_{LSS}\left(t\right)+\left(\lambda_{LS}\left(t\right)-\lambda_{D}\left(t\right)\right)\mu ,$$

(57) $$\dot{\lambda_{LI}\left(t\right)}=\left(\lambda_{LI}\left(t\right)-\lambda_{I}\left(t\right)\right)\gamma A_{LII}\left(t\right)+\left(\lambda_{LI}\left(t\right)-\lambda_{D}\left(t\right)\right)\mu ,$$

and

(58) $$\dot{\lambda_{D}\left(t\right)}=-C_{D}.$$

Furthermore, the end values of the adjoint variables all equal to 0, i.e.,

(59) $$\lambda_{S_{t_{f}}}=\lambda_{I_{t_{f}}}=\lambda_{A_{t_{f}}}=\lambda_{LS_{t_{f}}}=\lambda_{LI_{t_{f}}}=0.$$

Finally, according to the Pontryagin Maximum Principle, the optimal strategies are obtained by

(60) $$H\left(t,X^{*}\left(t\right),\lambda \left(t\right),\mu^{*}\left(t\right),\nu \left(t\right)\right)\leq H\left(t,X^{*}\left(t\right),\lambda \left(t\right),\mu^{*}\left(t\right),\nu^{*}\left(t\right)\right)\leq H\left(t,X^{*}\left(t\right),\lambda \left(t\right),\mu \left(t\right),\nu^{*}\left(t\right)\right).$$

□

As a consequence, in the optimal case, when $\left(\lambda_{I}\left(t\right)-\lambda_{S}\left(t\right)\right)\alpha_{1}S\left(t\right)I\left(t\right)>0$, the malware exerts the maximum effort to infect vulnerable sensor nodes; otherwise, it does not propagate. When $(\lambda_{D}\left(t\right)-\lambda_{I}\left(t\right))aI\left(t\right)>0$, the malware exerts the maximum effort to launch the hardware attack; otherwise, it does nothing in hardware equipped in sensor nodes. When $\left(\lambda_{I}\left(t\right)-\lambda_{LI}\left(t\right)\right)\gamma LI\left(t\right)+C_{C}\gamma LI\left(t\right)<0$, the malware exerts the minimum effort to influence the charging process to LI nodes; otherwise, the LI nodes accept the charging requests. Moreover, when $\left(\lambda_{A}\left(t\right)-\lambda_{I}\left(t\right)\right)\alpha_{2}I\left(t\right)<0$, WRSNs exist the maximum effort to clear the malware; otherwise, the networks do nothing in activating anti-malware program. When $\left(\lambda_{S}\left(t\right)-\lambda_{LS}\left(t\right)\right)\gamma LS\left(t\right)+C_{C}\gamma LS\left(t\right)<0$, WRSNs exist the maximum effort to charge the LS nodes; otherwise, LS nodes do not be charged.

## 4. Simulation

The purpose of this section is to further verify and develop the theorems stated in Section 3. In detail, the first three subsections focus on the stability of the system (1)–(6) and the last three subsections focus on the optimal control of the system (39)–(44).

The parameters used in the simulations were set as: Λ=0.2, $\alpha_{1}=0.0001$, $\alpha_{2}=0.001$, $\beta_{1}=0.005$, $\beta_{2}=0.005$, $\beta_{3}=0.008$, μ=0.004, a=0.005, and γ=0.05. All simulations were run on MacOS Catalina (Intel Core i5, 8GB, 1.8GHz) and MATLAB 2017b.

### 4.1. Stable Analysis When $R_{0}<1$

**Figure 3:**
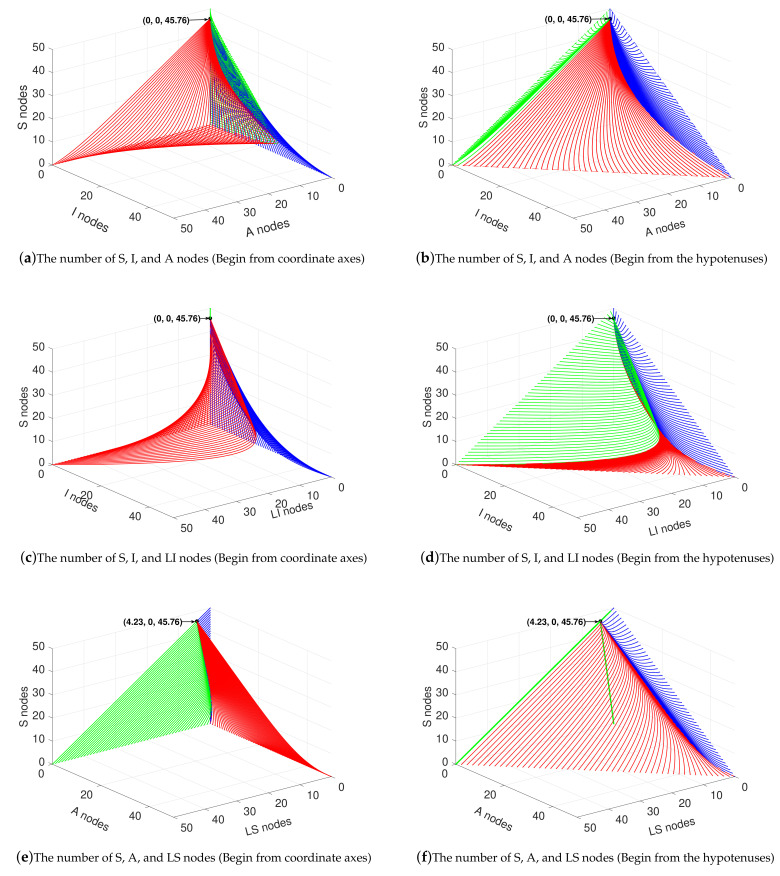
Variation of state variables in the case of $R_{0}<1$.

In this subsection, the stability of the system (1)–(6) is verified when $R_{0}<1$. Substituting the parameters into (19), we obtained $R_{0}=0.432<1$. Thus, there must exist a disease-free equilibrium point $\left(S_{0},I_{0},A_{0},LS_{0},LI_{0}\right)$ in the system. According to (10), $S_{0}=45.76$, $I_{0}=0$, $A_{0}=0$, $LS_{0}=4.23$, and $LI_{0}=0$. The simulation results are illustrated in Figure 3.

For the purpose of showing the changing trend of the system in a more three-dimensional and comprehensive way, we consider to verify the stability of the system in the form of three dimensions. We set N(t)≤50 (i.e., S(t)+I(t)+A(t)+LS(t)+LI(t)≤50). Therefore, in the case of three dimensions, the feasible region is a regular triangular pyramid with an equilateral triangle at its base and a right-angled isosceles triangle (Waist = 50) at its three sides.

The curves in Figure 3a,c,e all begin from the axes and the curves in Figure 3b,d,f all start at the boundary on the hypotenuses.

As shown in Figure 3a,b, in the three-dimensional area formed by the number of *A* nodes as the x-axis, the number of *I* nodes as the y-axis, and the number of *S* nodes as the z-axis, the curves eventually converge to (0,0,45.67) from the six boundaries. In detail, in Figure 3a, when the curves start from x-axis, it is assumed that that there exists only *A* and LI nodes in the networks at the initial moment; when the curve starts from the z-axis, it is assumed that that only *S* and LI nodes in the network at the initial moment; and when the curve starts from y-axis, it is assumed that that only *I* and LS nodes in the network at the initial moment. The purpose of these assumptions is to ensure that malware exists in the network at the beginning, otherwise it would be meaningless. In Figure 3b, in the *S*-*A* plane, we set the sum of *S* nodes and *A* nodes as 49, and the number of LI nodes as 1 at the beginning. In the *S*-*I* plane, we set the sum of the number of *S* and *I* nodes as 50 at the beginning. In the *A*-*I* plane, we set that the sum of the number of *A* and *I* nodes is 50 at the beginning.

Similarly, in the three-dimensional area formed by the number of LI nodes as the x-axis, the number of *I* nodes as the y-axis, and the number of *S* nodes as the z-axis, the curves eventually converge to (0,0,45.67), as shown in Figure 3c,d. Here, the principle of assumption is the same as above. The curves start from y-axis contain only *I* and LS nodes at the beginning. The curves start from the x-axis initially contain only LI and LS nodes at the beginning. It is worth noting that, in Figure 3c, the curve starts from the z-axis is reunited with the z-axis because it does not contain malware at the beginning. In Figure 3d, the curves start from the *S*-LI plane initially contain only *S* and LI nodes; the curves start from the *S*-*I* plane initially contain only *S* and *I* nodes; and the curves start from the *I*-LI plane initially contain only *I* and *LI* nodes.

In the three-dimensional area formed by the number of LS nodes as the x-axis, the number of *A* nods as the y-axis, and the number of *S* nodes as the z-axis, the curves eventually converge to (4.23,0,45.76), as shown in Figure 3e,f. Similarly, in Figure 3e, the curves begin from the z-axis initially contain *S* and *I* nodes; the curves begin from the x-axis initially contain LS and *I* nodes; and the curves begin from the y-axis initially contain *A* and *I* nodes. In Figure 3f, the curves begin from *S*-*A* plane initially contain *S*, *A*, and *I* nodes, and the sum of the number of *S* and *A* nodes are 49 and the number of *I* nodes is 1; the curves begin from *S*-LS plane initially contain *S*, LS, and *I* nodes, and the sum of the number of *S* and LS nodes are 49 and the number of *I* nodes is 1; and the curves begin from *A*-LS plane initially contain *A*, LS, and *I* nodes, and the sum of the number of *A* and LS nodes are 49 and the number of *I* nodes is 1.

In Figure 3a–d, when the initial number of *I* nodes is less than a threshold, the number of *I* nodes has a peak value and decreases after that, and finally reaches 0. When the number of *I* nodes is greater than this threshold, the number of *I* nodes decreases continuously because the number of newly infected nodes is smaller than the number of newly recovered nodes. All these results confirm Theorems 1 and 2.

### 4.2. Stable Analysis when $R_{0}>1$

**Figure 4:**
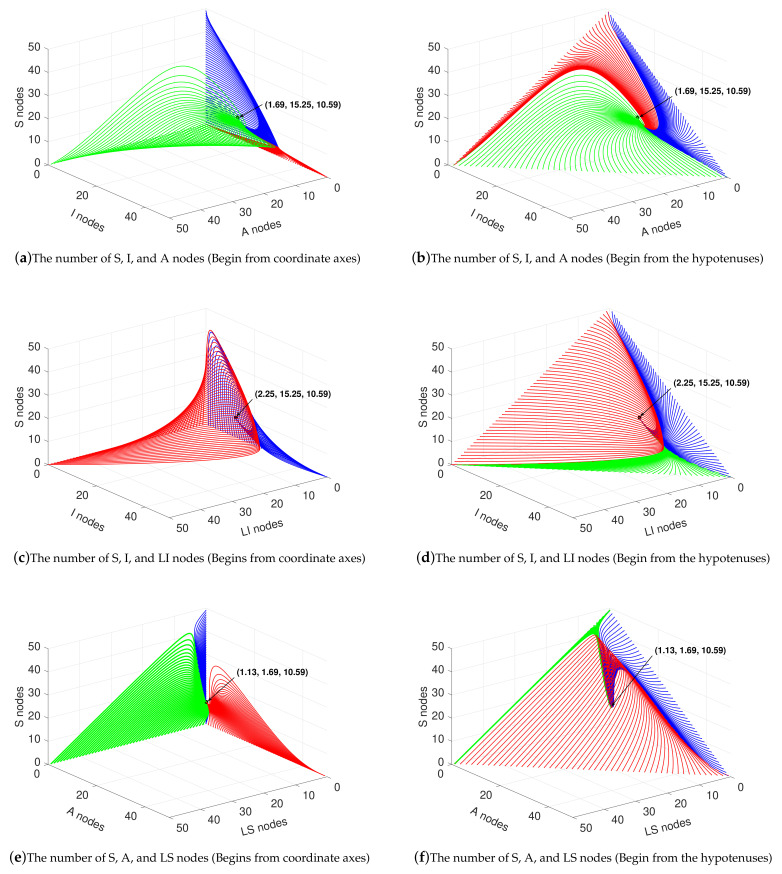
Variation of state variables in the case of $R_{0}>1$.

In this subsection, the situation under $R_{0}>1$ is discussed. Except for $\alpha_{1}=0.001$, the parameters remain the same as above. In this simulation, $R_{0}=4.320>1$, $S^{*}=10.59$, $I^{*}=15.25$, $A^{*}=1.69$, $LS^{*}=1.13$, $LI^{*}=2.25$ and N(∞)=30.9375 based on (10)–(13) and (19). As in the Section 4.2, suppose N(t)≤50. The simulation results are shown in Figure 4.

The assumptions at the initial moment of the curve in this subsection are the same as in Section 4.1. As shown in Figure 4a,b, in the three-dimensional area formed by the number of *A* nodes as the x-axis, the number of *I* nodes as the y-axis, and the number of *S* nodes as the z-axis, the curves eventually converge to (1.69,15.25,10.59) from the boundaries at the axes and the hypotenuses. In the three-dimensional area formed by the number of LI nodes as the x-axis, the number of *I* nodes as the y-axis, and the number of *S* nodes as the z-axis, the curves eventually converge to (2.25,15.25,10.59) from the boundaries, as shown in Figure 4c,d. In the three-dimensional area formed by the number of LS nodes as the x-axis, the number of *A* nodes as the y-axis, and the number of *S* nodes as the z-axis, the curves eventually converge to (1.13,1.69,10.59) from the boundaries, as shown in Figure 4e,f. All these results confirm Theorems 3 and 4.

Compared with the case of $R_{0}<1$, more peaks exist in the process of quantity change when $R_{0}>1$, but the general trend is similar. For *I* nodes, when the initial number is less than a certain threshold, it peaks and then eventually stabilize at the steady state value. When the initial number is greater than this threshold, the number of *I* nodes continues to decline until the steady state value. It is worth noting that the trend of the number of nodes is affected by the initial value. The trend changes if the initial values are set differently. However, if the model parameters do not change, the final value of the number of nodes does not change.

### 4.3. Influence of Parameters under Stable State

In this subsection, the influence of parameters on the spread of malware is analyzed. In detail, we analyzed the influence of $\alpha_{1}$, $\alpha_{2}$, $\beta_{3}$, and γ on the number of I nodes. The values of $\alpha_{1}$, $\alpha_{2}$, and $\beta_{3}$ range from 0.0001 to 0.01, and the value of γ ranges from 0.01 to 1.

**Figure 5:**
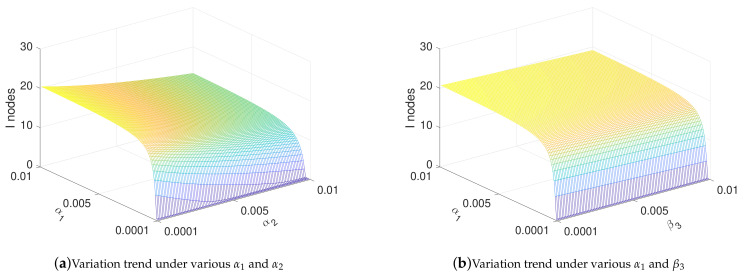

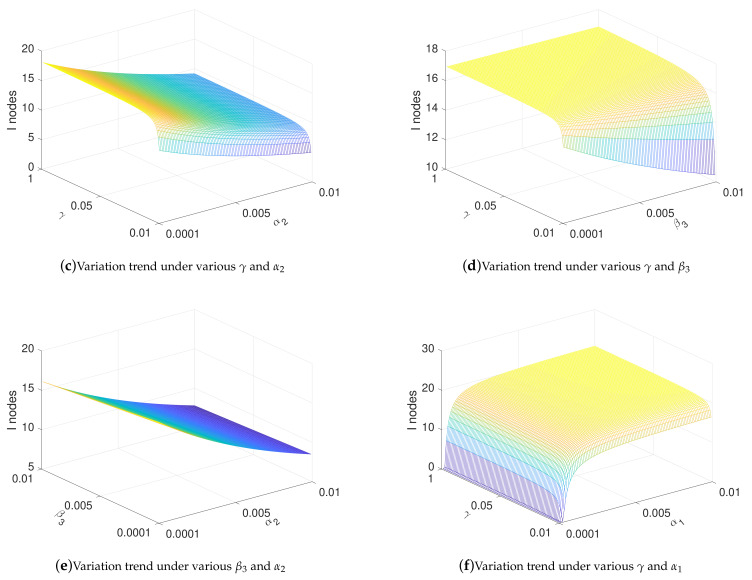
Change of infection under various parameters when t→∞.

Figure 5a shows the relationship between $\alpha_{1}$ and $\alpha_{2}$ and the number of *I* nodes when t→∞. Figure 5a shows that, by reducing the transmission rate $\alpha_{1}$, malware can eventually be cleared. At the same time, increasing the removal rate $\alpha_{2}$ of malware can effectively suppress the increasing of malware; Figure 5b shows the relationship between $\alpha_{1}$ and $\beta_{3}$ and the number of *I* nodes when t→∞. As shown in Figure 5b, the behavior of malware to drops nodes to LI state by increasing the frequency or intensity of exhaustion attacks cannot be too effective to increase the number of *I* nodes in the steady state. Figure 5c shows the relationship between γ and $\alpha_{2}$ and the number of *I* nodes in steady state. Figure 5c clearly shows that controlling the frequency or power of charging γ can restrain the spread of malware to a certain extent. Figure 5d shows the relationship between γ and $\beta_{3}$ and the number of *I* nodes in the steady state. As shown in Figure 5d, increasing the intensity of software attacks has little effect on the eventual prevalence of malware. On the contrary, when the charging rate γ drops to a certain extent, the amount of malware is greatly reduced. This suggests that we can control the charging rate γ to suppress the spread of malware. Figure 5e shows the relationship between $\beta_{3}$ and $\alpha_{2}$ and the number of *I* nodes in the steady state. In Figure 5e, the influence of $\beta_{3}$ on the eventual prevalence of malware is verified again. At the same time, the effect of increasing the rate of activating anti-malware programs on the prevalence of malware is more obvious. Figure 5f shows the relationship between $\alpha_{1}$ and γ and the number of *I* nodes in the steady state. As shown in Figure 5f, the method of reducing the number of *I* nodes by reducing the charging rate and transmission rate is verified again.

Among them, the most effective suppression method is to reduce the transmission rate $\alpha_{1}$. By increasing removal rate $\alpha_{2}$ and reducing the charging rate γ, the number of malware can be reduced to a certain extent when t→∞. In detail, although the method of reducing the transmission rate has a good effect, the effect is obvious when it is reduced to a certain extent, which is impractical in real life. The most direct method is to activate the anti-malware program to remove its own malware. The method of charging control is similar to the method of adjusting the transmission rate, which needs to be reduced to a certain threshold before the effect becomes obvious. Therefore, the method of suppressing malware by adjusting the transmission rate and charging rate is effective but requires much more consideration than activating the ant-malware program.

### 4.4. Variation of State Variables when $R_{0}<1$

Here, the evolution of state variables under optimal control is discussed. To verify the optimality, a non-optimal control group is set to compare with the optimal one. In detail, the situation under $R_{0}<1$ is stated first.

**Figure 6:**
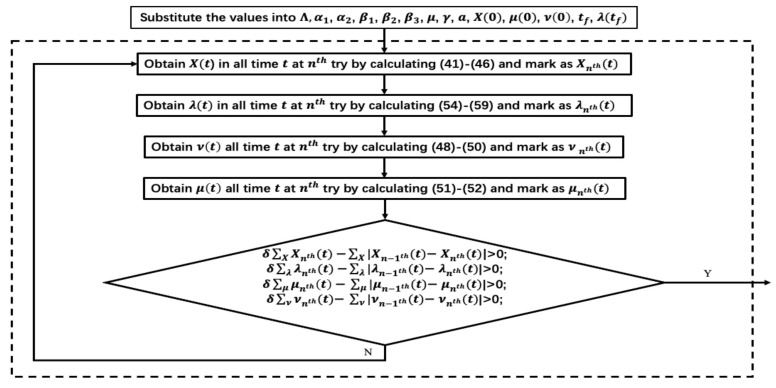
Flow diagram of Forward–Backward Sweep (FBS) method.

To satisfy (53)–(59), a Forward–Backward Sweep (FBS) method is applied. The flow diagram of the method is illustrated in Figure 6. First, the supposed values of model parameters are given. Then, by applying the finite difference method, the numerical solutions of the state variables are calculated in order and adjoint variables in reversed order. Furthermore, the values of controls are obtained at the same time. Finally, if and only if the difference between the two iterations is less than an error value δ multiplied by the iteration value at the current moment, then the optimality conditions stated in Theorem 5 are considered to be satisfied. Here, we set δ=0.001. It is worth noting that, when the system has low computational complexity, the FBS method can achieve better convergence of the adjoint variables. However, with the increasing complexity of the system, the method has difficulty achieving convergence, and it needs to update, which is also one of the directions of our future work.

**Figure 7:**
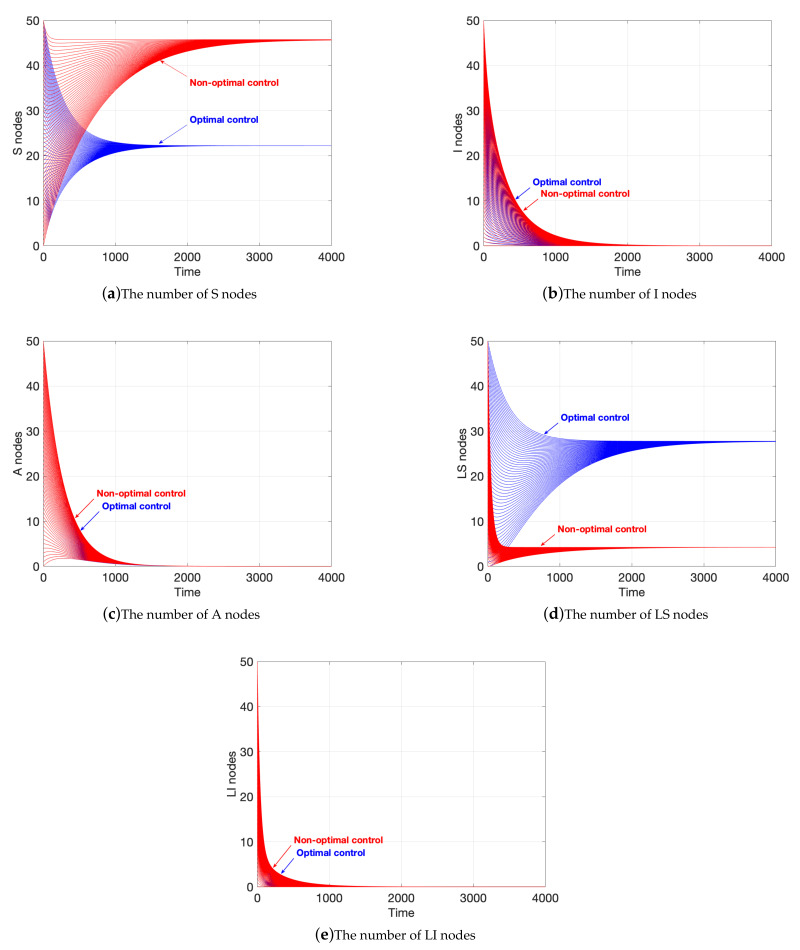
Evolution of state variables under various controls in the case of $R_{0}<1$.

Figure 7 shows the comparison of evolution of state variables under optimal control and non-optimal control. Here, the blue lines represent the evolution under optimal control and the red lines represent the evolution under non-optimal control. In Figure 7a, we set up 100 datasets, in which cases of the optimal control and the non-optimal control are equally divided. In the sets under optimal control, we assume that the sum of the initial number of nodes *S* and *I* of the networks is 50. For example, when the initial number of *S* nodes is 24, the initial number of *I* nodes is 26. Figure 7a shows the comparison of the number of *S* nodes in the two cases. Figure 7a shows that the number of *S* nodes under optimal control reach the equilibrium point more quickly, and the number is less than that under non-optimal control. Furthermore, the number of *S* nodes under optimal control is lower than that under non-optimal control when the number stay steady.

The data in Figure 7b follow those in Figure 7a. In the case of optimal control, as the number of *S* nodes decreases, the number of *I* nodes decreases more rapidly, as shown in Figure 7b. In other words, malware is eliminated faster under optimal control.

In the setting of the data of the two cases in Figure 7c, we assume that it contains *A* and *I* nodes at the beginning, and the sum is 50. As illustrated in Figure 7c, the difference in the number of *A* nodes is not evident in the two cases, which indicates the removal action never stops.

In Figure 7d, we assume that it contains only LS and LI nodes at the beginning, and their sum is 50. As illustrated in Figure 7d, the reason for the decrease in the number of *S* nodes is that WRSNs choose to stop charging the LS nodes, which leads to an increase to the number of LS nodes.

The data setting in Figure 7e follows that in Figure 7d. Similarly, the difference in the number of LI nodes is not significant in the two cases, as shown in Figure 7e, which indicates the software attacks never stop.

Therefore, although the spread of malicious programs can be suppressed under the optimal control, the performance of the system is sacrificed, that is, the existence of more low-energy sensor nodes leads to problems in network operation.

### 4.5. Variation of State Variables when $R_{0}>1$

In this subsection, the situation under $R_{0}>1$ is discussed. Comparing with Section 4.4, if the value of *T* is too high, the adjoint variables do not converge finally under the FBS method, so we set the terminal time of the game to 200, i.e., T=200. Meanwhile, the data setting is the same as in Section 4.5.

**Figure 8:**
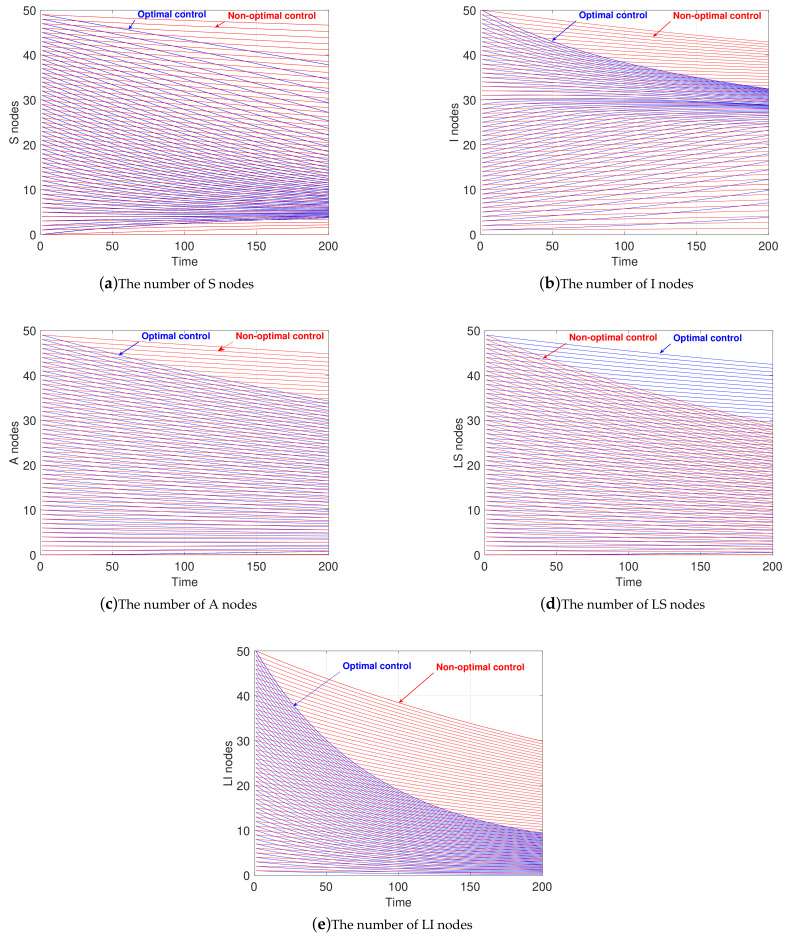
Evolution of state variables under various controls in the case of $R_{0}>1$.

Figure 8 shows the comparison of the changes in the number of the state variables in the same two cases stated in Section 4.4. Similar to the statement in Section 4.4, the number of *S* nodes under optimal control always shows a faster decline, as shown in Figure 8a. In contrast, the number of *S* nodes with non-optimal control does not change much from time 0 to 200. Therefore, in the case of $R_{0}>1$, WRSNs can restrain the growth of *I* nodes by reducing the number of *S* nodes.

Compared with the case without optimal control, more LS nodes stay in the *LS* state at this time instead of returning to the *S* state, as shown in Figure 8d. For the case of non-optimal control, the number of LS nodes decreases rapidly since LS nodes constantly send charging requests and get fully charged. For the optimal control, LS nodes choose to stop charging in order to reduce the growth rate of *S* nodes’ number.

As illustrated in Figure 8b,c,e, with the effective reduction of the number of *S* nodes, the numbers of *I* nodes, *A* nodes, and LI nodes all show a significant decrease, compared with the situation under non-optimal control.

In the case of $R_{0}>1$, for malware, to make the cost as large as possible, the three means controlled by malware maintain the maximum degree of control; for WRSNs, in addition to removing malware in the maximum efforts, it also stops charging the LS nodes to further deter more vulnerable nodes from being attacked.

### 4.6. Influence of Parameters under Optimal Controls

As in Section 4.3, the influence of parameters on malware is developed here. It is easy to know from Section 4.4 that, when $R_{0}<1$, malware is completely eliminated eventually. Therefore, we only consider the case $R_{0}>1$. At the same time, to maintain the continuity with Section 4.3, suppose T=200.

**Figure 9:**
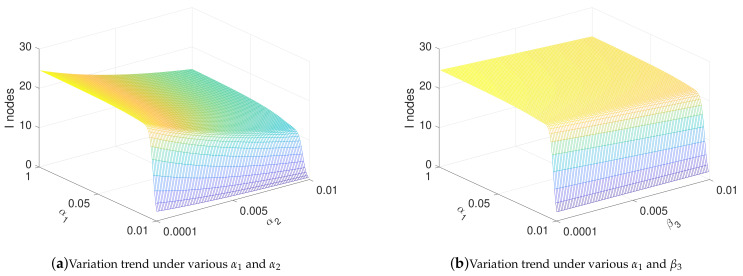

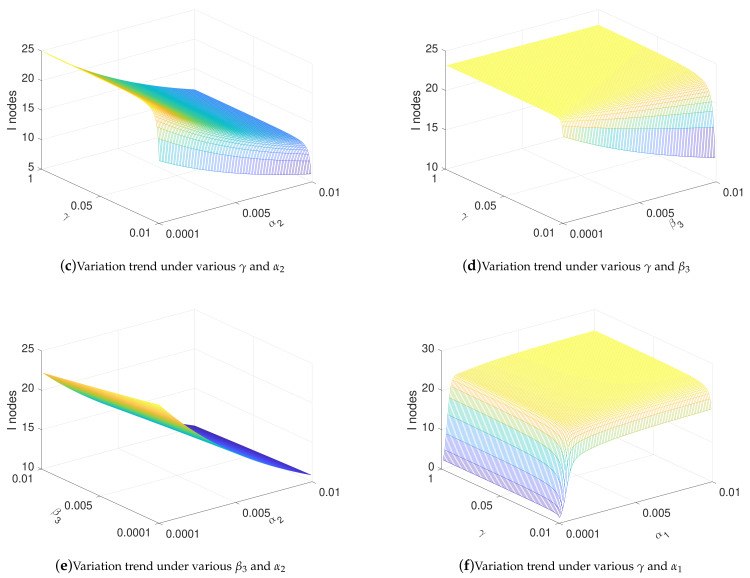
Change of infection in optimal controls under various parameters when t = 200.

Figure 9 shows the influence of the parameters on the number of *I* nodes, and the range of the parameters is consistent with Section 4.3. It is worth mentioning that, at this time, since the time setting is much smaller than that in Section 4.3, the number of *I* nodes is larger. Comparing with Figure 5 in Section 4.3, it is not difficult to find that, under optimal control, the influence of parameters on the propagation of malware is very similar to that under non-optimal control. Similarly, the conclusion is similar to Section 4.3, and is not repeated here.

In the optimal dynamic game, the three control methods of malware, namely $A_{SI}\left(t\right)$, $A_{ID}\left(t\right)$, and $A_{LII}\left(t\right)$, are always present and undiminished. As WRSNs, it stops charging the LS nodes while exerting greatest effort to activate the anti-malware program. Therefore, in the game process, the overall architecture of SIALS model is not affected. In other words, stopping charging has little effect on the model. Meanwhile, this phenomenon also reveals that the influence of reduced charging rate on the spread of malware mainly occurs in the state transition of sensor nodes from LI state to *I* state.

## 5. Conclusions

In this paper, we use epidemiology to propose a dynamic model, namely SIALS, describing the propagation of malware in WRSNs. In this model, not only the remaining energy of the sensor nodes is revealed, but also the description of the recovered process is enriched by introducing the anti-malware (*A*) state. Meanwhile, through the stability analysis of the model, we proved the local and global stability of disease-free equilibrium point and the epidemic equilibrium point. Furthermore, based on the confrontational nature of malware and WRSNs, this paper proposes a five-tuple attack–defense game model. Specifically, after introducing the overall cost, by adopting the Pontryagin Maximum Principle, this paper introduces the dynamic optimal strategies for malware and WRSNs. We verified the validity of the theories through simulations in the form of three-dimensional figures and analyzed the influence of the parameters on the propagation of malware. Then, the evolution of the number of state variables based on optimal control in the two cases of $R_{0}<1$ and $R_{0}>1$ was also simulated and analyzed. Meanwhile, the influence of parameters on infection under optimal control was analyzed.

Simulation results show that the malware can be eliminated by adjusting the transmission rate, but it needs to be reduced to a certain threshold. Activating anti-malicious program is the most effective and direct way to suppress the spread of malware. Adjusting the charging rate can also suppress the spread of malware effectively, but, again, it needs to be below a certain threshold. In the dynamic game between malware and WRSNs, WRSNs effectively reduce the number of malware by refusing to charge. In particular, in the case of $R_{0}<1$, malware goes extinct more quickly. In the case of $R_{0}>1$, the spread of malware is suppressed obviously compared with the case with non-optimal control.

With the continuous development of the wireless power transfer and the intelligent mobile vehicles, the potential security risk of mobile charger cannot be ignored. In our future work, in view of the integrating of various devices, both the homogenous and heterogenous cases will be taken into consideration, and, if the ability permits, the stochastic modeling and the advanced mathematical theories will be applied. Consequently, we hope our works can give some inspirations to interested researchers.

## Figures and Tables

**Table 1:** Research on WRSN security.

Authors	Problems	Methods	Results
A.N. Nguyen et al. [12]	Securing the physical layer	Time-switching power-splitting (TSPS) mechanism	The secrecy performance under TSPS is higher than the traditional scheme
J. Jung et al. [13]	Excessive energy consumption in the forward error correction(FEC) method	Energy-aware FEC method	The developed method performs better than the former one.
V.N. Vo et al. [14]	Securing energy harvesting wireless sensor networks(EH-WSNs) under eavesdropping and signal interception	An optimization scheme that uses a wirelessly powered friendly jammer	The hypotheses are supported.
A. EI Shafie et al. [15]	Securing a single-antenna rechargeable source node in the presence of a multi-antenna rechargeable cooperative jammer and a potential single-antenna eavesdropper	An efficient scheme which can optimize the transmission times of the source node	The average secrecy rate gain of the scheme is demonstrated significantly
B. Bhushan et al. [16]	Securing the mobile sinks position information	Energy Efficient Secured Ring Routing (E2SR2) protocol	E2SR2 achieves improved performance than the existing protocols
S. Lim et al. [17]	Securing EH-WSNs under the Denial-of-Service (DoS) attacks	Hop-by-hop Cooperative Detection (HCD) scheme	HCD scheme can significantly reduce the number of forwarding misbehaviors and achieve higher packet delivery ratio
K J.S.R. Kommuru et al. [18]	Balancing the trade-off between improving security and reducing energy consumption	Low complexity XOR technique and Hybrid LEACH-PSO algorithm	The proposed approach performs better than the existing approaches.
A. DI Mauro et al. [19]	Securing the communications under energy constraints	Adaptive approach which allows nodes to dynamically choose the most appropriate parameters	Adaptive solution performs better
X. Hu et al. [20]	Securing the up-link (UL) transmission	Establishing the communication model; deriving the energy outage probabilities (EOP), connection outage probabilities (COP) and secrecy outage probabilities (SOP) through comprehensive analysis	The theoretical derivations are verified
O. Bouachir et al. [21]	Securing the transmission between sensor nodes and base stations	A novel strategy to select cluster heads and implement the non-orthogonal multiple access (NOMA) technique in the transmission	The secrecy performance can be improved

**Table 2:** Research on stability of epidemic model in WSNs.

Authors	Characteristics	Model	Stability
S.Y. Huang et al. [22]	Heterogeneity	Susceptible-Infected-Quarantined- Recovered-Susceptible (SIQRS)	1
P.K. Srivastava et al. [23]	Anti-malware process	Susceptible-Exposed-Infectious- Antimalware-Recovered (SEIAR)	2
L.H. Zhu et al. [24]	Time delay	Susceptible-Believed-Denied (SBD)	2
G.Y. Liu et al. [25]	Low-energy	Susceptible-Infected-Low-energy-Susceptible(SILS)	1
S. Hosseini et al. [26]	User awareness, network delay and diverse configuration of nodes	Susceptible–Exposed–Infected–Recovered-Susceptible with Vaccination and Quarantine state	2
R.P. Ojha et al. [27]	Quarantine and vaccination techniques	Susceptible–Exposed–Infectious–Quarantined–Recovered–Vaccinated (SEIQRV)	2
D.W. Huang et al. [28]	Patch injection mechanism	Susceptible–Infected–Patched–Susceptible (SIPS)	3
L.H. Zhu et al. [29]	Time delay in homogeneous and heterogeneous networks	Ignorants–Spreaders1–Spreaders2–Stiflers1–Stiflers2 (I2S2R)	1
J.D. Hernández Guillén et al. [30]	Carrier state	Susceptible–Carrier–Infectious–Recovered–Susceptible (SCIRS)	1
S.G. Shen et al. [31]	Heterogeneity and Mobility	Vulnerable–Compromised–Quarantined–Patched–Scrapped (VCQPS)	2

1: Local and global stability in malware-free and epidemic points; 2: Local and global stability in malware/rumor/worm-free point; 3: local and global stability in epidemic point.

**Table 3:** Research on differential game applied in WSNs.

Authors	Players	Goal	Strategies
S. Eshghi et al. [32]	Malware and mobile WSNs	Leverage the heterogeneity of malware propagation	Optimal patching policies
M.H.R. Khouzani et al. [33]	Malware and Mobile WSNs	Attain desired tradeoffs between security risks and bandwidth consumption	Optimal control in activating dispatchers and selecting their transmission rate
L.T. Zhang, et al. [34]	Malware and device to Device (D2D) offloading-enabled mobile network	Understand the malware propagation process in D2D offloading-enabled mobile network	Optimal dynamic defense and attack strategies
H. Al-Tous et al. [35]	An energy-harvesting multi-hop WSN	Balance the normalized buffer states of all sensor nodes and minimize the amount of energy used for data transmission.	An online power control and data scheduling algorithm
Y.H. Huang et al. [36]	Virus and sensor nodes	Mitigate virus spreading	Virus-resistant weight adaptation policies
Y. Sun et al. [37]	Edge nodes (ENs)	Realize the balance between reward and energy consumption cost of ENs in the deployment of defense measures	Optimal defense strategy
S.G. Shen et al. [38]	malware and WSNs	Limit malware in WSNs	Optimal dynamic strategies for the system and malware
J.H. Hu et al. [39]	A healthcare-based wireless sensor network (HWSN)	Minimize the transmission cost	Optimal data transmission strategies
S. Sarkar et al. [40]	Multi-hop wireless networks	Optimize network throughput	Optimal routing and scheduling policies

**Table 4:** Epidemiological coefficients of the model.

Symbol	Description
Λ	Birth rate
γ	The rate of charging sensor nodes from low-energy to high-energy
$\beta_{1}$	Depletion rate determined by the working strength of susceptible nodes
$\beta_{2}$	Depletion rate determined by the working strength of anti-malware nodes
$\beta_{3}$	Depletion rate determined by malware
$\alpha_{1}$	Transmission rate of malware
$\alpha_{2}$	The rate of activating anti-malware
μ	Death rate
*a*	The rate of hardware attack determined by malware

## Data Availability

The data presented in this study is contained within the article.
